# Male Reproductive Organ Weight: Criteria for Detection of Androstenone-Positive Carcasses in Immunocastrated and Entire Male Pigs

**DOI:** 10.3390/ani13122042

**Published:** 2023-06-20

**Authors:** Gregor Fazarinc, Nina Batorek-Lukač, Martin Škrlep, Klavdija Poklukar, Alice Van den Broeke, Kevin Kress, Etienne Labussière, Volker Stefanski, Milka Vrecl, Marjeta Čandek-Potokar

**Affiliations:** 1 Veterinary Faculty, University of Ljubljana, Gerbičeva ulica 60, 1000 Ljubljana, Slovenia; gregor.fazarinc@vf.uni-lj.si (G.F.); milka.vrecl@vf.uni-lj.si (M.V.); 2 Agricultural Institute of Slovenia, Hacquetova ulica 17, 1000 Ljubljana, Slovenia; nina.batorek@kis.si (N.B.-L.); martin.skrlep@kis.si (M.Š.); klavdija.poklukar@kis.si (K.P.); 3 ILVO (Flanders Research Institute for Agriculture, Fisheries and Food), Scheldeweg 68, 9090 Melle, Belgium; alice.vandenbroeke@ilvo.vlaanderen.be; 4 Department of Behavioral Physiology of Livestock, Institute of Animal Science, University of Hohenheim, Garbenstraße 17, 70599 Stuttgart, Germanyvolker.stefanski@uni-hohenheim.de (V.S.); 5 PEGASE, INRAE, Institut Agro, 35590 Saint-Gilles, France; etienne.labussiere@inrae.fr; 6 Faculty of Agriculture and Life Sciences, University of Maribor, Pivola 10, 2311 Hoče, Slovenia

**Keywords:** boar taint, morphometric indicators, immunocastration, entire males

## Abstract

**Simple Summary:**

Alternatives to surgical castration of piglets present a challenge for control of boar taint. In this study, the proportion of animals with androstenone levels above the risk threshold for boar taint (>0.5 µg/g fat) was found to be less than 5% in immunocastrated animals (ICs), and testicular weight was a reliable indicator of androstenone-positive carcasses. In contrast, in the entire males (EMs), the proportion of animals with androstenone levels above the risk threshold was much higher (approximately 80%), and the difference in testicular size between EMs with androstenone levels below and above the risk threshold was less pronounced. However, the pelvic urogenital tract weight was reliable in these animals. We thus propose that pelvic urogenital tract and testes weights may be considered a simple, reliable, and time- as well as cost-efficient morphometric indicator for the identification of androstenone-positive carcasses for both ICs and EMs at the slaughter line.

**Abstract:**

Immunocastration and rearing of entire males (EMs) are sustainable alternatives to surgical castration. However, these animal carcasses have variable risk of boar taint and should be identified at the slaughter line. We aimed to identify a simple and reliable indicator of androstenone-related boar taint by evaluating pelvic urogenital tract weight as a marker of boar-taint animals at the slaughter line. The pelvic urogenital tract, testes, and accessory sex glands of EMs and immunocastrates (ICs) were collected, dissected, and weighed, before colorimetric measurements of testicular tissue. Additionally, GnRH antibody titers and testosterone, androstenone, and skatole levels were determined. Our results showed that 81.8% of EMs had androstenone levels above the risk threshold (>0.5 µg/g fat; EM/A^high^ subgroup), whereas in ICs, the C/A^high^ subgroup with androstenone >0.5 µg/g fat accounted for only 4.3%. Androstenone levels correlated negatively with GnRH antibody titers and positively with testosterone levels and reproductive organ weights. Identification of ICs with androstenone levels above the threshold (IC/A^high^ subgroup) may be achieved via testes or pelvic urogenital tract weight measurements. However, in EMs, the latter is a more reliable parameter. A principal component analysis based on these variables and hierarchical clustering also distinguished the A^high^ from the A^low^ subgroup, irrespective of IC/EM. The findings highlight the possible use of pelvic urogenital tract weight along with testes weight as a simple, reliable, and efficient morphometric indicator for identifying androstenone-positive carcasses of different sex categories.

## 1. Introduction

Meat from uncastrated male pigs may have an undesirable odor and taste, referred to as boar taint that has been ascribed to high levels of androstenone, a metabolite of testosterone and dihydrotestosterone [1] and skatole [2]. Boar taint may be prevented by castrating young male piglets within the first week postpartum without anesthesia, as per EU legislation. Surgical castration, however, is painful and stressful for the animal, carries a risk of infection, and leads to decreased growth performance as well as increased mortality. Consequently, the process is unproductive from the standpoint of animal welfare and economic gain [3]. Additionally, surgically castrated pigs tend to be fatter and have poorer feed conversion, which is reflected in increased production costs [4]. An alternative approach to surgical castration is the administration of an anti-gonadotropin-releasing hormone (GnRH) vaccine that has been available in the EU since 2009. Vaccination induces a specific antibody- mediated response that neutralizes endogenous GnRH, leading to stoppage of testicular development. The blocked hypothalamic–pituitary axis inhibits release of pituitary gonadotropic hormones, namely luteinizing hormone (LH) and follicle-stimulating hormone (FSH), and inhibits testicular steroid synthesis. This finally results in the regression of reproductive organs, inhibition of testicular androstenone production, and the consequent elimination of the compounds responsible for undesirable pork odor. A decrease in androstenone levels is also followed by a decrease in the levels of skatole as a result of free hepatic metabolic activity due to lowered testicular steroid levels [5]. To achieve an effect, the vaccine is administered twice, with the second vaccination usually administered 4–6 weeks prior to slaughter in order to improve feed conversion and lean meat gain in males [6].

Although the GnRH vaccine Improvac^®^ reduces testicular endocrine activity, immunization may rarely be ineffective, with 0.7 to 4.0% cases of non-responders reported [7,8,9]. Additionally, immunization may be unsuccessful due to non-responsiveness owing to disease, escape from immunization during mass vaccination on farms, or improper vaccination [7,10,11]. The carcasses of these animals are therefore at high risk of boar taint and should be identified at the slaughter line [12].

Identification of tainted entire males (EMs) in the slaughter line is a serious challenge [13]. As demonstrated in a recent systematic review, up to 56.0% of EM carcasses have more than 1 μg/g androstenone, up to 34.0% have more than 0.2 μg/g skatole, and up to 17.0% of EMs are classified as tainted via sensory evaluation [14].

Tainted meat can be used in processed meat products, where boar taint is masked by adding spices, curing, smoking, or mixing with untainted meat [14]. Quantification of fat, androstenone, and skatole levels, the primary compounds responsible for boar taint, is time-consuming and expensive. A rapid and reliable indicator is thus required for the identification of boar- taint-positive animals at the slaughter line. While reproductive organ size may be useful in this context, opinions about the most suitable organ that should be analyzed to detect androstenone-dependent boar taint carcasses are variable. While the evaluation of testicular size has been proposed as a suitable indicator, Bonneau [15] suggested that accessory sex gland size, especially that of vesicular glands, is a more reliable indicator of effective immunization against boar taint. Recent studies have also demonstrated that colorimetric measurements of testicular parenchyma differ between immunocastrated pigs (ICs) and EMs, since the testes of the former are paler, more yellow, and less red as compared to that of the latter [16,17].

The present study aimed to determine if reproductive organ size may serve as a reliable indicator for the identification of androstenone-positive animals at the slaughter line. Toward this end, testes and accessory sex glands of EMs and ICs were collected at the slaughter line, dissected, and weighed, followed by colorimetric measurements of testicular cross-sections. Additionally, a comparative analysis between the weight of the complete pelvic urogenital tract and previously evaluated indicators, including testicular and accessory sex gland size, was performed to verify its suitability for the detection of carcasses with high androstenone levels. In addition, principal component analysis of the variables studied and subsequent hierarchical clustering based on the principal components were performed to assess their suitability for classifying pigs in terms of androstenone content and associated boar taint risk.

## 2. Materials and Methods

### 2.1. Animals

Within the scope of project SuSI (Sustainability in pork production with immunocastration), a total 135 EMs and 282 ICs, commercial crosses, mainly with Pietrain, from different selection programs (different countries) were enrolled in the study. ICs were vaccinated according to the standard protocol, i.e., with 2 mL Improvac^®^ (Pfizer Animal Health, Louvain-la-Neuve, Belgium), first at 12 weeks of age and again 4 to 5 weeks prior to slaughter.

Entire males (EMs) and ICs were subdivided into two groups based on androstenone level: pigs with adipose tissue androstenone levels above the threshold of boar taint risk (A^high^), and pigs with androstenone levels below the threshold (A^low^). The androstenone level threshold for boar taint risk was set at 0.5 µg/g fat [18,19].

### 2.2. Blood and Fat Samples Analysis

Blood samples were collected from pigs right before slaughter or at exsanguination at the slaughter line to determine antibody response using a specific anti-GnRH binding assay, and testosterone levels were measured using the in-house RIA methods, as described previously [20]. Plasma removed post centrifugation was stored at −20 °C until further analysis. All concentrations were expressed in ng/mL plasma, and the limit of detection for testosterone was 0.24 ng/mL plasma. 

Androstenone and skatole concentrations were measured in back fat samples using HPLC as previously described by Batorek et al. [6]. The concentrations were expressed in μg/g liquid fat, and the detection limits for androstenone and skatole were 0.24 μg/g and 0.03 μg/g, respectively.

### 2.3. Reproductive Organ Measurement

The success rate of vaccination against GnRH was tested by weighing reproductive organs. Pelvic reproductive organs were excised and weighed at the slaughter line, as previously described [21]. Briefly, the entire pelvic part of the urogenital tract with the urinary bladder was removed from the pelvic cavity and separated from the rectum and anus. The urinary bladder was emptied via an incision made at its apex, and the pelvic urogenital tract was cleared of excess adipose and connective tissue. The penis was cut at the caudal end of the bulbourethral glands. The pelvic urogenital tract comprising the accessory reproductive glands, namely the vesicular and bulbourethral glands as well as the prostate, emptied bladder, and pelvic urethra, was weighed. Subsequently, the accessory reproductive glands were dissected and weighed. The testes with epididymides were detached from the vaginal tunic and the spermatic cord, which was cut at the level of the head of the epididymis. Both the testes and epididymides were weighed together for each animal.

### 2.4. Colorimetric Analysis

Colorimetric measurements L*, a*, and b* (Commission Internationale d’Eclairage) denoting lightness, redness, and yellowness of the testicular tissue, respectively, were assessed on cross-section of the left testis using a Minolta Chroma Meter CR-300 (Minolta Co., Ltd., Osaka, Japan) with an 11 mm aperture and D65 illuminant, calibrated against a white tile. To avoid apparatus bias, the colorimetric values obtained from different consortium partners were normalized in the range of 0–1 before statistical analysis.

### 2.5. Statistical Analyses

Statistical analyses were performed using SigmaPlot 12.5 (Systat Software, Inc., Erkrath, Germany). Based on sex category, i.e., ICs or EMs, and androstenone status, namely A^high^ or A^low^, the groups were defined as IC/A^low^, IC/A^high^, EM/A^low^, and EM/A^high^. Differences between groups were analyzed using one-way analysis of variance and making pairwise multiple comparisons using Tukey’s test. Differences were considered statistically significant at *p* < 0.05.

The Pearson correlation coefficient test was used to analyze the relationship between reproductive organ weight and androstenone and skatole levels. Correlation coefficients (r) with values (+ or −) between 0.4 and 0.69, 0.7 and 0.89, and above 0.9 were considered to be indicative of moderate (^+^), strong (^++^), and very strong correlations (^+++^), respectively [22].

In addition, principal component analysis (PCA) was performed. This is a descriptive tool with no need for rigorous distributional or model assumptions, implying that it can be used on a wide range of data, which can diverge considerably from the ‘ideal’ of multivariate normality [23]. To confirm that weights of reproductive organs and testicular colorimetric values could be used to detect risky animals, PCA was performed with R package FactoMiner [24] including testes, urogenital tract and accessory reproductive glands weight and testes color parameters followed by hierarchical clustering on principal components (HCPC). Missing values for urogenital tract accessory sex gland weight and testis parenchyma color were imputed with a PCA. Means for each variable were scaled before the implementation of missing data imputation with a regularized iterative PCA method using R package missMDA [25]. Uncertainty regarding the predictions of missing data was assessed with MIPCA analysis. Thereafter, the functions PCA and HCPC of package FactoMineR [24] were used. The first two components of PCA had an eigenvalue greater than 1, which is the recommended threshold for use in hierarchical clustering. Considering the shape of the tree and the bar graph of inertia gain [24], two clusters were determined, which represent animals with low (<0.5 µg/g fat; A^low^) and high (>0.5 µg/g fat; A^high^) androstenone.

## 3. Results

### 3.1. Boar Taint Compounds and Plasma Concentrations of Testosterone and GnRH Antibodies

Immunization of male pigs against GnRH using commercial Improvac^®^ vaccine proved effective in controlling testicular androstenone production. Vaccination considerably reduced the number of pigs with androstenone levels in fat that were above the threshold for boar taint risk (0.5 µg/g). In the EM group, 81.8% of pigs had androstenone levels above the risk threshold (EM/A^high^ subgroup), while the IC/A^high^ subgroup accounted for only 4.3% of the IC group (Table 1). In general, androstenone levels in fat were approximately eight-fold lower in the IC group than those in the EM group. Further, carcasses in the EM/A^high^ subgroup were significantly heavier than those in the other subgroups. Interestingly, differences in skatole concentration were observed only between the IC/A^low^ and EM/A^low^ subgroups. Additionally, while testosterone levels were significantly higher in the IC/A^high^ than that in the IC/A^low^ subgroup, they were similar in both EM subgroups. As expected, a much higher percentage of GnRH- binding antibodies were detected in the plasma of IC pigs than those in EM, and in IC/A^low^ than in IC/A^high^ pigs (Table 1).

### 3.2. Reproductive Organ Weights and Colorimetric Analysis of Testicular Tissue

The data on reproductive organ weight and testicular tissue colorimetric analysis as summarized in Table 2 revealed that vaccination against GnRH successfully inhibited reproductive organ development. The testicular weight in the IC group was two to three times lower than that in the EM group, and it was higher in the A^high^ than that in the A^low^ subgroup within each sex category. Similar weight differences were evident for the vesicular and bulbourethral gland but not for the prostate and urogenital tract. Weight regression was most pronounced in the vesicular gland, and least in the bulbourethral gland.

The results of testicular colorimetric analysis were indicative of differences between the experimental groups (representative photo for differences between sexes are provided in Supplementary Figure S1). In general, the testicular tissues of EMs had lower L*, higher a*, and lower b* values than those of ICs, thus implying that the testes of EM were darker, more reddish, and less yellow than those of the ICs. The testes of the IC/A^high^ subgroup were also significantly darker than those of the IC/A^low^ subgroup as was evident from lower L* values. The colorimetric values were not significantly different in the A^high^ and A^low^ subgroups within the EMs.

### 3.3. Correlation Analysis

The correlations between reproductive organ weights, boar taint compounds, GnRH antibody titers, and testosterone levels are presented in Table 3. All reproductive organ weights showed moderate to strong positive correlation with androstenone levels, with the highest degree of correlation found for the urogenital tract. On the contrary, no such trends were observed for skatole. As expected, the weights of all reproductive organs correlated strongly and negatively with GnRH antibody titers, as well as moderately and positively with plasma testosterone concentrations. Testicular lightness (L*) correlated negatively with androstenone and testosterone levels, and positively with GnRH antibody titers.

### 3.4. A Principal Component Analysis

**Figure 1:**
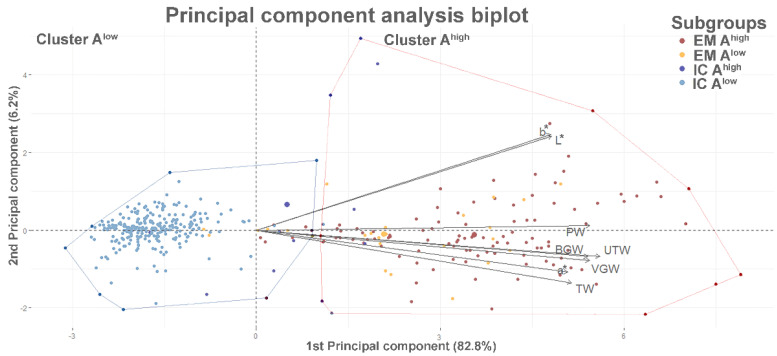
Principal component analysis (PCA) showing the distribution of boars across all four experimental subgroups (entire males (EMs) and immunocastrated pigs (ICs) with low (A^low^) and high (A^high^) androstenone concentrations; *n* = 417) according to the first two principal components of PCA of selected variables (*n* = 8) presented in Table 2. The biplot of the first two principal components jointly represents 89% of the total variation. Selected variables strongly correlated with PC1 are indicated using arrows. GTW—genital tract weight; TIW—testes weight; PW—prostate gland weight; BGW—bulbourethral gland weight; VGW—vesicular gland weight; L*—lightness, a*—redness, and b*—yellowness of testicular parenchyma. The clusters determined by hierarchical clustering on principal components are indicated in different color lines, i.e., A^low^ [blue] vs A^high^ [red]. Note individual misclassified pigs (2 false positive and 5 false negative cases in ICs; 10 false positive and 12 false negative cases in EMs).

Next, we tested whether it was possible to classify pigs with respect to boar taint, i.e., androstenone content, using only the variables described in Table 2. A principal component analysis biplot (Figure 1) showed that the first two components of PCA had eigenvalues >1 and together accounted for 89% of the variation. The first principal component (PC1) alone accounted for 82.8% and should help to reveal trends between androstenone-positive (A^high^) and androstenone-negative carcasses (A^low^). Indeed, two clusters associated with the first two dimensions of PCA (Figure 1) showed good separation between A^high^ and A^low^ carcasses. However, the correctness of assignment to the A^low^ or A^high^ cluster was much better for the ICs (two or 0.7% false positive and five or 1.8% false negative cases) whereas for EMs, we could observe ten (7.4%) false positive and twelve (8.9%) false negative cases.

## 4. Discussion

Our results confirmed that vaccination against GnRH effectively controls testicular androstenone production. This was evident from the observation that only 12 of 282, i.e., 4.26% ICs, had androstenone concentrations above the risk threshold (0.5 µg/g fat; IC/A^high^). Immunocastration also resulted in regression of all reproductive organs, albeit to varying extents. The reproductive organs, especially the testes of ICs with levels of androstenone > 0.5 µg/g fat (subgroup IC/A^high^) were almost twice as large as those in the IC/A^low^ subgroup. These pigs had 10 to 20 times higher GnRH antibody titers than those in the EM/A^low^ and EM/A^high^ subgroups, respectively. However, their anti-GnRH antibody titers were significantly lower than those in the IC/A^low^ subgroup. It is possible that the IC/A^high^ subgroup had positive immune responses to vaccination that did not reach the critical antibody levels required to suppress testicular steroid production. Apparently, the antibody titer required to block testicular steroid production differs between successfully and unsuccessfully immunized animals within a narrow range, resulting in higher plasma testosterone and fat androstenone levels in the latter. Notably, suppression of Leydig cell function may only be assumed when the testosterone level is below the threshold of 0.5 ng/mL [26], which was not the case in the IC/A^high^ subgroup.

The extent of boar taint compound accumulation is an outcome of balance maintained between the synthesis and degradation of androstenone and skatole [13]. While we observed effective prevention of androstenone accumulation due to immunocastration, this was not the case for skatole levels. Backfat skatole concentrations were generally low and were similar in ICs and Ems, which does not support previous observations showing a strong positive correlation between fat androstenone and skatole concentrations [17,27]. This can be explained by the fact that skatole is produced by bacterial breakdown of tryptophan in the large intestine, and its production is influenced by diet, gut microflora, and the quantity of mucosal debris. Blood skatole levels are proportional to skatole production in the gut and differ between pigs that are fed the same diet [28]. Immunocastration should lower fat skatole levels since the lowering of androstenone levels significantly reduces CYP2E1 and CYP2A activities, which are crucial for hepatic phase 1 skatole metabolism [29]. Immunocastration is also believed to reduce plasma IGF-1 levels, which slows mucosal cell turnover in the large intestine, and consequently decreases skatole production [30,31]. We did not observe any correlation between skatole and androstenone concentrations, even in EMs, where the liver degrading enzyme is likely to be metabolically burdened with high androstenone levels. This could be a consequence of good hygiene, health, and housing conditions because animals were all raised in experimental facilities with reinforced cleaning protocols when compared to commercial housing conditions [32]. A recent study suggested that there is no relationship between fatty acids constituting the fat and skatole and androstenone contents [33].

Fat androstenone levels correlated negatively with GnRH antibody titers and positively with testosterone levels and reproductive organ weight. We thus hypothesized that reproductive organ weight may be a suitable criterion for the detection of androstenone-positive carcasses on the slaughter line. The correlation analysis showed that total pelvic urogenital weight correlates strongly with androstenone concentration and, therefore, may be a reliable indicator for discriminating between EMs with high or low androstenone levels. On the other hand, testicular weight should be used to evaluate the success of immunocastration. The accessory sex glands are very sensitive to reduced testicular activity, resulting in rapid recession, especially of the vesicular gland, which regresses immediately post successful immunocastration. The watery secretions of the vesicular gland are readily reabsorbed or drained, causing it to shrink up to sevenfold in immunocastrated pigs. The shrinkage of the bulbourethral gland was less pronounced due to its thicker muscle wall and highly viscous contents. The vesicular gland is thus a reliable indicator of immunocastration success; however, based on a previous report, it does not always allow correct classification of EMs, since its regression is not necessarily accompanied by regression of the bulbourethral gland and/or testes [34]. The size of the vesicular gland varies widely in EMs, and its delicate structure may result in potential leakage of contents during excision, making it unsuitable for routine measurements. The bulbourethral gland is anatomically easy to identify and manipulate due to its firm structure and highly vicious content; however, its lesser and slower regression lowers the success rate in distinguishing between ICs and EMs. The porcine prostate is relatively small, partially covered by a vesicular gland, and is therefore anatomically unsuitable for routine measurements.

Androstenone levels are directly influenced by testicular steroidogenic enzyme activity. Our results show that testes and pelvic urogenital weights are reliable indicators of immunocastration success and of androstenone-related boar taint in carcasses (Table 1). Immunocastrates with testes’ weight greater than approximately 450 g (Table 2) should be considered as ‘at androstenone risk’ on the slaughter line. This is further supported by colorimetric measurements of testicular cross-sections, particularly the L* values, which showed a strong negative correlation with fat androstenone levels. In general, while the testicular tissue of EMs and IC/A^high^ pigs was darker (L*) than that of IC/A^low^ pigs, the other color parameters (a* and b*) were not sufficiently reliable to distinguish between A^high^ and A^low^ pigs within the sex category subgroups. In particular, L* (lightness) is an appropriate parameter for detecting IC testes with high androstenone levels (IC/A^high^), but not in EMs. As previously reported [35], the colorimetric analysis could be a suitable additional screening tool if properly standardized and appropriate color charts are developed for the detection of androstenone-positive ICs.

Due to the lack of societal acceptance, immunocastration is not a suitable solution in several countries where other alternatives, including rearing EMs, are employed. Slaughter at lower age and weight is a possible strategy to reduce boar taint risk in EMs [19]. Another possible approach is to sort boar taint carcasses and use them in different types of meat products to effectively mask the taint [36]. More than 80% of EMs in our study had androstenone levels above the risk threshold (EM/A^high^ subgroup). They simultaneously exhibited heavier testes and pelvic urogenital tracts, which may therefore be used as possible indicators of androstenone-positive EM carcasses. The difference in urogenital tract weights between EM/A^high^ and EM/A^low^ were substantially higher (~30%) than those observed for the testes (~15%). Removal of the pelvic urogenital tract is relatively simple and does not require precise anatomical dissection of accessory glands, so it can be performed in slaughter- line conditions. The higher weight difference of the urogenital tract compared to testes also helps in limiting the effect of imperfect dissection. Even though the testes are more accessible, they should be removed from the scrotum and vaginal tunic. Additionally, testicular weight differences between EM/A^high^ and EM/A^low^ pigs are low, thus making it a less reliable indicator of high androstenone levels in EMs than the pelvic urogenital tract. As for the vesicular, prostate, and bulbourethral glands, they require precision during excision and manipulation, thus rendering them unsuitable for routine manipulation under slaughter- line conditions. The PCA with hierarchical clustering further supported our observation, as it was possible to distinguish androstenone-positive carcasses (A^high^) from those with an androstenone levels below the boar taint risk threshold (A^low^) with high accuracy especially in IC pigs.

## 5. Conclusions

The present study demonstrates drastic suppression of testicular androstenone synthesis using vaccination against GnRH in male pigs. Additionally, fat androstenone levels were found to correlate well with reproductive organ weights. A small percentage of ICs with high androstenone levels were easily identified in the slaughter line via their testes, which were approximately two times heavier than those of the remaining animals. In EMs, testicular size differences between pigs with high and low androstenone levels were small, and accurate identification of EMs with high androstenone levels thus requires weighing the pelvic urogenital tract, comprising all the accessory glands. Colorimetric analysis of the testes, especially lightness, revealed differences between ICs with androstenone levels above threshold, but not in EMs. In summary, testicular and pelvic urogenital tract weight may be employed as a simple, reliable, and time- as well as cost-efficient morphometric indicator for the identification of androstenone-positive carcasses in both ICs and EMs at the slaughter line.

## Figures and Tables

**Table 1:** Carcass weight, fat skatole concentrations, plasma testosterone levels, and anti- GnRH antibodies in immunocastrated pigs (IC group), as well as in entire males (EM group) and with low (A^low^) and high androstenone concentration (A^high^).

	IC Group		EM Group	
Subgroup	IC/A^low^	IC/A^high^	EM/A^low^	EM/A^high^
Number of pigs	270	12	24	108
Carcass weight (kg)	86.8 ± 0.7 ^a^	82.4 ± 2.0 ^a^	80.6 ± 3.1 ^a^	91.5 ± 1.1 ^b^
Androstenone (µg/g fat)	0.24 ± 0.01 ^a^	0.95 ± 0.19 ^c^	0.31 ± 0.02 ^b^	3.60 ± 0.41 ^d^
Skatole (µg/g fat)	0.10 ± 0.01 ^a^	0.12 ± 0.02 ^ab^	0.15 ± 0.04 ^b^	0.13 ± 0.02 ^ab^
Testosterone (ng/mL plasma)	0.48 ± 0.06 ^a^	3.20 ± 0.96 ^b^	49.99 ± 19.90 ^c^	45.65 ± 4.77 ^c^
GnRH antibody binding (%)	45.46 ± 0.41 ^c^	38.73 ± 4.02 ^b^	2.13 ± 0.13 ^a^	3.72 ± 0.26 ^a^

Data are presented as mean ± SEM; A^high^ androstenone level > 0.5 µg/g of fat; A^low^ androstenone level < 0.5 µg/g of fat. Values within rows with different superscripts are significantly different (*p* < 0.05; one-way analysis of variance followed by Tukey’s pairwise multiple comparisons test).

**Table 2:** Weights of reproductive organs and testicular colorimetric values in immunocastrated pigs (IC group) and entire males (EM group) with low (A^low^) and high (A^high^) androstenone concentrations.

	IC Group		EM Group	
Subgroup	IC/A^low^	IC/A^high^	EM/A^low^	EM/A^high^
Reproductive organ weight (g)				
* Testes	246.57 ± 0.10 ^a^	454.17 ± 241.97 ^b^	587.30 ± 32.43 ^c^	696.81 ± 13.87 ^d^
Vesicular gland	23.38 ± 21.00 ^a^	52.80 ± 17.35 ^b^	123.73 ± 21.31 ^c^	223.37 ± 14.13 ^d^
Prostate	2.37 ± 0.13 ^a^	2.40 ± 0.71 ^a^	5.18 ± 0.59 ^b^	7.70 ± 044 ^c^
Bulbourethral gland	39.39 ± 1.72 ^a^	71.45 ± 14.85 ^b^	91.10 ± 10.74 ^b^	148.67 ± 6.20 ^c^
Urogenital tract	163.25 ± 5.98 ^a^	184.47 ± 32.94 ^a^	407.33 ± 112.67 ^b^	597.50 ± 24.28 ^c^
^#^ Testes cross-sectional surface color				
Lightness (L*)	55.53 ± 3.25 ^a^	52.76 ± 3.60 ^b^	46.08 ± 1.18 ^b^	47.22 ± 0.58 ^b^
Redness (a*)	14.32 ± 0.18 ^a^	16.64 ± 0.95 ^ab^	17.42 ± 0.84 ^b^	17.98 ± 0.28 ^b^
Yellowness (b*)	12.63 ± 0.27 ^a^	12.26 ± 1.03 ^a^	11.90 ± 1.25 ^b^	10.58 ± 0.47 ^b^

Data are presented as mean ± SEM; A^high^ androstenone level > 0.5 µg/g of fat; A^low^ androstenone level < 0.5 µg/g of fat. * Weight of the right and left testes along with the epididymis. ^#^ Normalized values in the range 0.0–1.0 were used for statistical analysis. Values within rows with different superscripts are significantly different. (*p* < 0.05; one-way analysis of variance followed by Tukey’s pairwise multiple comparisons test).

**Table 3:** Correlation (Pearson’s correlation coefficients) between reproductive organ data (weights and testes’ colorimetric values), boar taint compounds (androstenone and skatole), GnRH antibody levels, and testosterone levels in all subgroups.

	Androstenone	Skatole	GnRH Antibody Titer	Testosterone
Testes	0.515 ^+^	0.162	−0.848 ^++^	0.684 ^+^
Vesicular glands	0.703 ^++^	0.054	−0.834 ^++^	0.724 ^++^
Prostate	0.649 ^++^	0.081	−0.830 ^++^	0.569 ^+^
Bulbourethral glands	0.663 ^++^	0.043	−0.813 ^++^	0.645 ^+^
Urogenital tract	0.749 ^++^	0.024	−0.864 ^++^	0.726 ^++^
L*	−0.485 ^+^	−0.182	0.543 ^+^	−0.513 ^+^
a*	0.278	0.163	−0.346 ^+^	0.321
b*	−0.138	−0.268	0.334 ^+^	−0.247

Values with ^++^ denote a strong correlation and values with ^+^, a moderate correlation between the two variables. L*—lightness, a*—redness and b*—yellowness of testicular parenchyma.

## Data Availability

The data that support the findings of this study are available from the corresponding author upon reasonable request.

## Supplementary Materials

The following supporting information can be downloaded at: https://www.mdpi.com/article/10.3390/ani13122042/s1, Figure S1: The cross section of testes from immunocastrated (A) and entire (B) male pig made for measurement of color in CIE L*, a*, b* color space.
