# Supplementary material for: Male Reproductive Organ Weight: Criteria for Detection of Androstenone-Positive Carcasses in Immunocastrated and Entire Male Pigs
Source: Animals (Basel). 2023 Jun 20;13(12):2042. doi: 10.3390/ani13122042 (PMC10295219; doi:10.3390/ani13122042)
Supplement: Supplementary file 1 [file animals-13-02042-s001.zip › animals-2445235-supplementary.pdf]

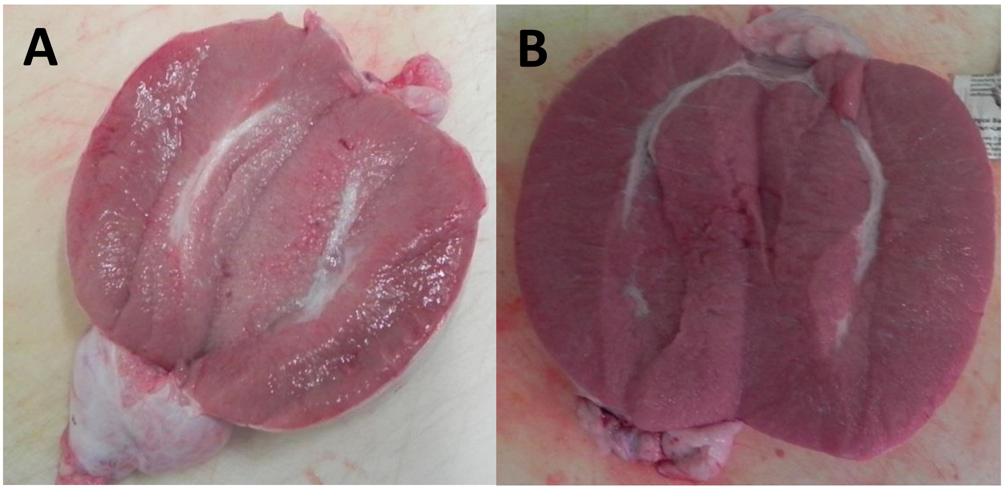

Figure S1: The cross section of testes from immunocastrated (**A**) and entire (**B**) male pig made for measurement of color in CIE L*, a*, b* color space.
